# Case Series: Single-port subxiphoid robotic excision of ectopic mediastinal parathyroid adenomas

**DOI:** 10.3389/fsurg.2026.1830874

**Published:** 2026-06-05

**Authors:** Shayan Hosseinzadeh, Alexander Chang, Mahsa Shariat, Nestor Villamizar

**Affiliations:** Department of Surgery, University of Miami Hospital, Miami, FL, United States

**Keywords:** da Vinci SP system, ectopic mediastinal parathyroid adenoma, primary hyperparathyroidism, robotic surgery, subxiphoid approach

## Abstract

**Background:**

Ectopic mediastinal parathyroid adenomas are a rare cause of persistent primary hyperparathyroidism and present operative challenges due to proximity to major mediastinal structures. Robotic trans-subxiphoid resection using multi-port platforms has been described; however, experience with a true uniportal single-port robotic approach remains limited.

**Methods:**

We report a case series of two patients with biochemically confirmed primary hyperparathyroidism and imaging-localized anterior mediastinal parathyroid adenomas who underwent excision using a single-port subxiphoid robotic technique with the da Vinci SP system. Operative variables, intraoperative parathyroid hormone (PTH) response, perioperative outcomes, and pathology were analyzed.

**Results:**

Single-port subxiphoid robotic excision was completed successfully in 100% (2/2) of cases with no conversions and no intraoperative complications. Mean operative time was 150 min (170 and 130 min), and estimated blood loss was <50 mL in both cases. Critical structures, including the innominate vein and bilateral phrenic nerves, were clearly visualized and preserved. Intraoperative PTH levels decreased by >80% at 10 min post-excision in both patients, meeting Miami criteria. PTH decreased from 172 pg/mL to 22 pg/mL in one patient and from 296 pg/mL to 99 pg/mL, with subsequent normalization, in the other. Both patients were discharged on postoperative day one with normalization of ionized calcium at 1-week follow-up. Final pathology confirmed parathyroid adenoma in both cases.

**Conclusions:**

This series demonstrates the safety and feasibility of a true uniportal single-port subxiphoid robotic approach for ectopic mediastinal parathyroid adenomas and highlights its potential as a highly minimally invasive alternative for selected patients.

## Introduction

Ectopic parathyroid adenomas arise from abnormal embryologic migration, and failure to identify them can result in persistent or recurrent primary hyperparathyroidism. Inferior ectopic glands are most commonly located in the anterior mediastinum, while superior glands are often found near the tracheoesophageal groove (1). Ectopic mediastinal parathyroid adenomas present a surgical challenge, often necessitating transcervical, transthoracic, or transsternal approaches (1, 2).

Robotic surgery offers three-dimensional visualization, tremor filtration, and articulating instruments, enabling precise dissection in confined spaces. The da Vinci Single-Port (SP) robotic system represents the latest evolution in robotic platforms, designed specifically for true uniportal surgery (3–10). While robot-assisted trans-subxiphoid excision of ectopic parathyroid adenomas has been reported using multi-port configurations (1, 2), to our knowledge, the use of a single-port robotic approach for this indication has not been previously published. This series presents two cases of ectopic mediastinal parathyroid adenomas successfully resected using a single-port robotic technique. We highlight the technical feasibility, safety, and potential advantages of the subxiphoid SP approach for complex anterior mediastinal lesions.

## Case presentations

### Case 1

A 69-year-old female presented with biochemical and radiologic evidence of an ectopic parathyroid adenoma in the anterior mediastinum, localized anterior to the ascending aorta and inferior to the left innominate vein.

### Imaging

**Figure 1:**
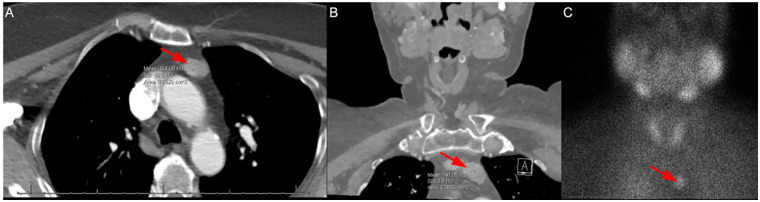
Axial **(A)** and coronal **(B)** CT images of the adenoma, with corresponding sestamibi scan **(C)** for the first case.

Parathyroid CT scan showed a 17.5 × 15 × 26 mm enhancing, mildly lobulated soft-tissue mass in the left anterior mediastinum abutting the left brachiocephalic vein. Imaging features were compatible with a possible ectopic parathyroid adenoma, although a nodal mass could not be excluded. The lesion showed no definite washout on delayed arterial phase and demonstrated overall lower attenuation than the thyroid on both early and delayed phases (Figures 1A,B).

On Sestamibi scan, there was expected early accumulation of the radiotracer in the thyroid gland with wash-out on delayed images. However, there was a soft tissue attenuating nodular-like lesion measuring 1.5 × 1.1 cm along the left paracentral upper anterior mediastinum with positive radiotracer accumulation on both early and delayed images, compatible with anterior mediastinum parathyroid adenoma (Figure 1C).

The patient's hyperparathyroidism was medically optimized preoperatively. After multidisciplinary discussion, a single-port subxiphoid robotic excision was planned.

### Surgical technique

**Figure 2:**
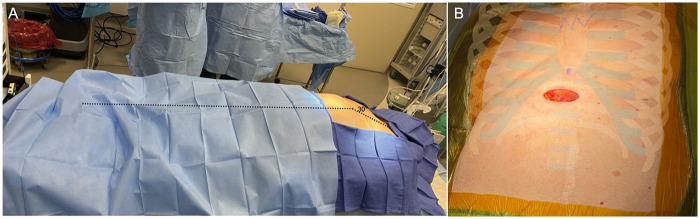
Patient positioned with table flexed at the xiphoid in 30° reverse trendelenburg **(A)** A 4 cm subxiphoid incision was created one fingerbreadth below the xiphoid **(B).**

**Figure 3:**
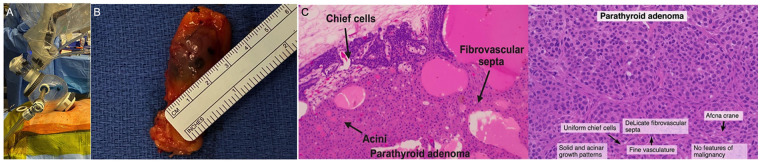
Single-port robotic system setup and positioning **(A)**, gross pathological features of the parathyroid adenoma **(B)**, and histopathologic examination of the parathyroid adenoma **(C).**

The patient was positioned supine with arms tucked and the table flexed at the xiphoid level in a 30° reverse Trendelenburg position (Figure 2A). A 4 cm transverse subxiphoid incision was made (Figure 2B), and the retrosternal space was bluntly dissected. A wound protector was inserted, and the da Vinci SP system was docked (Figure 3A).

Mediastinal Dissection: The anterior mediastinal pleura was incised medial to the internal mammary vessels, opening both pleural cavities. Pericardial fat was mobilized off the pericardium.

Identification of Critical Structures: Bilateral internal mammary veins, innominate vein, and bilateral phrenic nerves were identified and preserved.

Tumor Identification and Excision: Thymic horns were dissected from the innominate vein, and adipose tissue in the aortopulmonary window was mobilized until the ectopic adenoma was visualized. A draining vein was clipped and divided, and the mass was excised *en bloc* with adjacent adipose tissue.

Specimen Retrieval and Closure: The specimen was extracted via the wound protector, hemostasis was secured. The rectus sheath was reapproximated, the pneumothorax evacuated, and the incision closed in layers. Intraoperative parathyroid hormone (PTH) levels were measured pre-excision, and 10 min post-excision. Intraoperative PTH levels demonstrated a classic pattern of successful single-gland disease treatment, falling rapidly from a baseline of 172 pg/mL to 22.1 pg/mL at 10 min, representing an >85% reduction and reaching the normal range, and fulfilled the Miami criterion and confirmed biochemical cure after excision of a solitary adenoma.

### Postoperative course

She experienced an uncomplicated postoperative recovery and was discharged on postoperative day 1. At 1-week follow-up, the patient's ionized calcium had normalized to 1.2 mmol/L**,** improved from a preoperative level of 1.6 mmol/L.

### Pathologic findings

Gross examination revealed a 12.4-gram fibroadipose specimen with a well-circumscribed tan mass measuring 2.7 × 1.5 × 1.3 cm (Figure 3B). Microscopically, the lesion consisted of uniform chief cells in solid and acinar patterns, compatible with a parathyroid adenoma, without features of malignancy (Figure 3C).

### Case 2

A 38-year-old female presented with findings consistent with an ectopic parathyroid adenoma in the anterior mediastinum.

### Imaging

**Figure 4:**
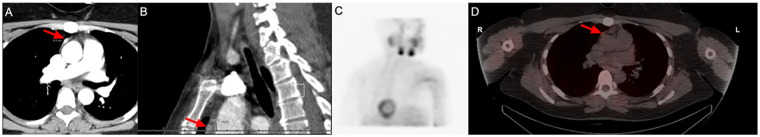
Axial **(A)** and sagittal **(B)** CT images of the adenoma, along with sestamibi scan **(C)** and SPECT **(D)**, for the second case.

Parathyroid CT demonstrated a 10 mm oval nodule in the anterior mediastinum adjacent to the ascending thoracic aorta, corresponding to uptake on prior SPECT-CT, most suggestive of ectopic parathyroid adenoma (Figures 4A,B).

On Sestamibi scan, there was no uptake in the neck or upper chest on early or delayed planar imaging (Figure 4C). However, SPECT-CT demonstrated a 0.7-cm soft tissue nodule in the anterior mediastinum, with mild radiotracer uptake, suspicious for an ectopic parathyroid adenoma in the anterior mediastinum, adjacent to the ascending thoracic aorta at the level of the right ventricular outflow tract (Figure 4D).

### Surgical technique

Position and initial dissection followed the same steps as described in Case 1.

Although the adenoma was not clearly visualized, all soft tissues corresponding to preoperative imaging were excised with an appropriate margin. Pericardial fat was dissected off the pericardium, thymic tissue mobilized off the innominate vein, and a few draining thymic veins were clipped. Both phrenic nerves were identified and preserved. Thymic and mediastinal adipose tissues were dissected off the aortopulmonary window and ascending aorta. The specimen was retrieved through the wound protector, hemostasis confirmed.

Intraoperative PTH levels initially decreased from 296 pg/mL pre-excision to 99 pg/mL at 10 min, meeting the percentage-drop requirement but remaining above the normal range, and then, after a slight rebound, eventually decreased appropriately to 50 pg/mL. At 1-week follow-up, the patient's ionized calcium remained normal at 1.14 mmol/L.

### Pathologic findings

Pathology examination revealed a hypercellular parathyroid gland and benign thyroid tissue, consistent with a parathyroid adenoma.

### Postoperative course

She experienced an uncomplicated postoperative recovery and was discharged on postoperative day 1.

## Discussion

This case series demonstrates the feasibility, safety, and technical precision of a single-port subxiphoid robotic approach for the resection of ectopic mediastinal parathyroid adenomas. The use of the da Vinci SP system provides direct midline access to the anterior mediastinum, excellent visualization of bilateral phrenic nerves, and avoidance of intercostal incisions, which may reduce postoperative pain compared with lateral thoracic approaches (10–15). Robotic articulation and three-dimensional visualization are particularly advantageous for dissection in proximity to critical vascular structures such as the innominate vein and ascending aorta (16).

Ectopic mediastinal parathyroid adenomas present unique surgical challenges that distinguish them from other anterior mediastinal lesions. These tumors are typically small, often difficult to localize intraoperatively, and frequently embedded within thymic or mediastinal adipose tissue. In addition, they are commonly located adjacent to critical vascular structures, including the innominate vein and aortopulmonary window. Unlike other mediastinal tumors, the primary goal of resection is biochemical cure rather than margin status, necessitating precise identification and complete excision of all hyperfunctioning tissue. These characteristics make surgical exposure, visualization, and meticulous dissection particularly important.

The single-port subxiphoid approach offers several disease specific advantages that directly address these challenges. First, the midline subxiphoid access provides a direct, symmetric view of the anterior mediastinum, allowing visualization of both phrenic nerves and facilitating bilateral exploration without repositioning. This is particularly advantageous for ectopic parathyroid adenomas, which may be located anywhere within thymic tissue or the aortopulmonary window. Second, the superior to inferior viewing angle enables more effective mobilization of thymic tissue and *en bloc* resection of surrounding mediastinal fat, which is often necessary given the variable and sometimes occult location of these glands. Third, the enhanced three-dimensional visualization and articulating instruments of the SP platform allow for precise dissection around delicate vascular structures, improving operative safety despite the small size and challenging location of these lesions. Finally, avoidance of intercostal access eliminates postoperative neuropathic pain, which is particularly relevant in cases involving small benign lesions where minimizing surgical morbidity is a priority.

Unlike previously described multiport trans-subxiphoid or intercostal robotic approaches (1, 2), this technique utilizes a true uniportal configuration without auxiliary incisions. Prior reports have demonstrated the feasibility of robotic resection of mediastinal parathyroid adenomas using multiport systems; however, these approaches often require intercostal access or additional ports to achieve adequate triangulation and exposure. In contrast, the single-port system enables all instruments and visualization to be delivered through a single subxiphoid incision, thereby preserving the benefits of minimally invasive surgery while maintaining adequate operative control.

From a technical standpoint, successful execution of this approach depends on careful docking strategy and optimization of the working environment. In our technique, the SP port is inserted through a subxiphoid incision with the robotic boom aligned in the midline to facilitate direct access to the anterior mediastinum. The remote center is positioned at the level of the fascial incision to minimize torque on the abdominal wall and maximize instrument articulation within the retrosternal space. A flexible three-dimensional camera is used in conjunction with articulating instruments, typically including a bipolar Maryland dissector, a bipolar grasper, and a fenestrated grasper, allowing precise dissection and exposure. CO₂ insufflation at 10 mmHg is used to develop and maintain the retrosternal working space, with lower pressures selected to balance adequate exposure with hemodynamic stability given the proximity to the heart and great vessels.

Operative times in this series averaged approximately 150 min, which is comparable to previously reported multiport robotic approaches (approximately 178 min) (17) and longer than conventional video assisted thoracoscopic surgery (VATS), which averages approximately 90 min (18). This likely reflects both the inherent technical demands of the single-port approach and the learning curve associated with adopting a new robotic platform. Notably, these cases were performed as part of the initial institutional experience with the da Vinci SP system in thoracic procedures. A couple cases of thymectomy for Myasthenia Gravis had been performed before the two cases of ectopic parathyroid adenoma described in this series. FDA approval for using the SP platform in thoracic surgery was obtained in July 2024. Our center was the 1st hospital in South Florida performing thoracic surgery with the robotic SP platform in October 2025. With increased experience and refinement of technique, operative efficiency is expected to improve.

Despite its advantages, the single-port subxiphoid approach presents several technical challenges. Instrument crowding, reduced triangulation, and limited working space through a single incision can make precise dissection more demanding, particularly around critical structures such as the innominate vein. In addition, the retrosternal working space can be restricted in patients with a deep chest or increased mediastinal adiposity, and cardiac motion may further complicate exposure and stability.

In clinical practice, these limitations can be mitigated through several strategies. Optimal patient positioning in reverse Trendelenburg allows gravitational displacement of mediastinal contents, improving exposure. Careful development of the retrosternal plane and early identification of key anatomical landmarks, including the innominate vein and bilateral phrenic nerves, are critical for maintaining orientation and safety. The use of articulating instruments inherent to the SP system helps compensate for reduced triangulation, while stable CO₂ insufflation enhances visualization and working space. With experience, these technical adaptations can significantly improve operative efficiency and safety.

Appropriate patient selection is essential to maximize the advantages of this approach. Ideal candidates include patients with small, well localized ectopic parathyroid adenomas located in the anterior mediastinum, particularly within thymic tissue or the aortopulmonary window. Patients without prior sternotomy or extensive mediastinal adhesions, and those with favorable body habitus allowing adequate subxiphoid access, are also well suited for this technique. Conversely, larger lesions, invasive pathology, or cases requiring complex vascular reconstruction may be better approached using multiport robotic or open techniques.

The intraoperative use of parathyroid hormone (PTH) monitoring was critical in confirming complete resection in both cases. In Case 2, although the PTH level did not immediately normalize, the observed >50% decline met Miami criteria, and subsequent normalization occurred postoperatively. This pattern is consistent with delayed biochemical normalization, which has been described in patients with single-gland disease despite appropriate intraoperative PTH decline, and underscores the importance of interpreting intraoperative values in clinical context.

Early postoperative outcomes in this series are consistent with prior reports of subxiphoid robotic surgery for anterior mediastinal pathology, which have demonstrated reduced postoperative pain, shorter hospital stay, and excellent surgical outcomes (19–22). Both patients in this series were discharged on postoperative day one without complications and achieved normalization of calcium levels. To further enhance the educational value and reproducibility of this technique, a supplementary intraoperative video demonstrating key procedural steps, including docking, exposure, identification of critical structures, and adenoma excision, has been included.

This study has several limitations. The sample size is small, consisting of only two patients, and the findings may not be generalizable. There is also potential selection bias, as both cases involved relatively small, favorably located lesions. In addition, long-term follow-up data are limited, and further studies with larger cohorts are needed to evaluate durability of outcomes and broader applicability. Despite these limitations, this series provides early evidence supporting the feasibility and potential advantages of the single-port subxiphoid robotic approach, serving as a foundation for future studies in larger patient populations.

## Conclusion

Single-port subxiphoid robotic surgery using the da Vinci SP system is a safe and feasible technique for the excision of ectopic mediastinal parathyroid adenomas. In carefully selected patients, it offers a highly minimally invasive approach that provides excellent visualization and precise dissection while minimizing surgical morbidity. Further studies are warranted to validate these findings and define its role relative to established surgical approaches.

## Data Availability

The original contributions presented in the study are included in the article/Supplementary Material, further inquiries can be directed to the corresponding author.
